# Exercise Prescription Enhances Maximal Oxygen Uptake and Anaerobic Threshold in Young Single Ventricle Patients with Fontan Circulation

**DOI:** 10.1007/s00246-021-02806-8

**Published:** 2022-02-01

**Authors:** Henri Pyykkönen, Otto Rahkonen, Nadja Ratia, Sini Lähteenmäki, Heikki Tikkanen, Päivi Piirilä, Olli Pitkänen-Argillander

**Affiliations:** 1 Faculty of Health Sciences School of Medicine, University of Eastern Finland, Kuopio, Finland; 2 Department of Pediatrics, Helsinki University Central Hospital and University of Helsinki, Helsinki, Finland; 3 Department of Internal Medicine, Helsinki University Central Hospital and University of Helsinki, Helsinki, Finland; 4 Unit of Clinical Physiology of the HUS Medical Diagnostic Center, Helsinki University Central Hospital and University of Helsinki, Helsinki, Finland

**Keywords:** Univentricular heart, Fontan circulation, Maximal oxygen uptake, Exercise prescription, Aerobic exercise

## Abstract

A modified Fontan procedure is performed to palliate single ventricle malformations. This hemodynamic arrangement sets systemic venous pressure unphysiologically high which predisposes the patient to severe long-term complications. As a means of self-care, exercise may ease transpulmonary flow. We investigated the effects of 6-month exercise prescription on pediatric Fontan patients. Eighteen stable Fontan patients (14 ± 2.6 years, 160.4 ± 11.3 cm, and 51.4 ± 14.4 kg) were recruited. Baseline fitness was assessed by physical activity questionnaire, body composition, cardiorespiratory performance, and muscle fitness tests. Exercise prescription was individually tailored for a 6-month training period at home. At entrance to the study, Fontan patients had lower than normal maximal oxygen uptake (VO_2max_) of 28. ± 5.9 ml/kg/min (61 ± 11% of normal). VO_2max_ significantly correlated with weekly amount of habitual exercise and muscle mass of the lower limbs (*p* < 0.001 for both). After 6 months of training, the patients had improved their anaerobic threshold of 18 ± 3.5 vs 20 ± 4.8 ml/kg/min, *p* = 0.007, and workload tolerance of 119 ± 39 vs 132.4 ± 44 W, *p* = 0.001. At EUROFIT tests, the patient muscle fitness was below age-matched reference, but correlations existed between VO_2max_ and lower limb muscle tests. Our patients with Fontan hemodynamics were able to positively respond to an exercise program by enhancing submaximal performance which should be beneficial for getting through daily activities. Future studies should correlate whether hemodynamic findings at Fontan completion influence physical activity and exercise reserves, and whether these predict predisposition to chronic complications.

## Introduction

Treatment of patients born with a univentricular heart necessitates a major stepwise palliative heart surgery where the goal is to connect the systemic veins directly into the pulmonary arteries and to use the single ventricle as a pump for the aorta. Although lifesaving, the Fontan procedure results in a pulmonary circulation very different from physiological conditions, with a progressive risk of attrition over time. Without the subpulmonary ventricle, the systemic venous pressure increases 2- to 3-fold in comparison with physiologic normal [1].

In addition, the lack of a pulsatile flow within the pulmonary arterial tree results in impaired pulmonary artery growth and increased pulmonary vascular resistance [2, 3]. As a consequence, increased pulmonary vascular resistance will lead to reduced ventricular preload and cardiac output. Very little can be done by pharmacological means to optimize the function of the successfully accomplished Fontan creation. The chronically increased systemic venous pressure will in most cases eventually lead to overt organ failure [4, 5].

Fontan-operated patients have frequently been described with limited exercise tolerance [6, 7] and investigations have been directed to elucidate whether regular exercise training could improve the patient’s well-being and hemodynamic function and improve their prognosis. Previous studies suggest that aerobic training and lower limb-focused strength training may be a beneficial way to improve the aerobic capacity of Fontan patients, but their significance to the outcome and functional well-being of the patient needs to be investigated [8, 9].

The aim of this study was to evaluate whether after a comprehensive baseline assessment, a personal exercise prescription would improve the aerobic performance of Fontan patients. We hypothesized that in single ventricle patients, maximal cardiopulmonary exercise capacity can be increased by regular aerobic training and bodyweight exercise focusing on lower limbs.

## Methods

### Study Design

**Fig. 1:**
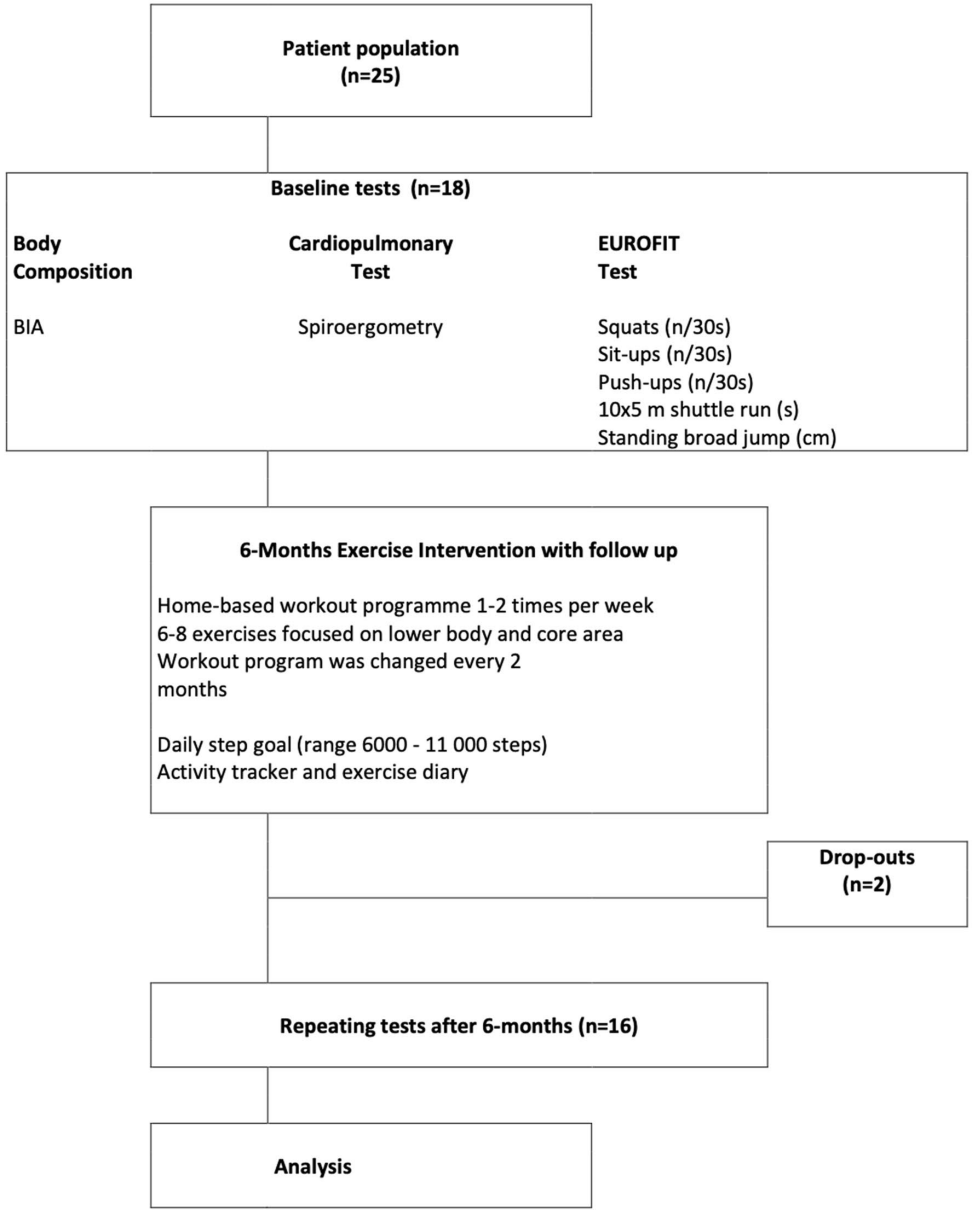
Study design of the 6-month intervention period. Baseline tests with method (*n* = 18). *BIA* bioelectrical bioimpedance analysis

We performed a prospective clinical trial evaluating the efficacy of 6-month exercise prescription for Finnish 8–17-year-old Fontan-operated patients taken care of at the ambulatory Pediatric University clinics in Finland. To mitigate anatomical bias, Fontan patients with both left ventricle and right ventricle as the systemic pump were enrolled. In addition, all patients needed to be self-acting and without any obvious end-organ injury. Exclusion criteria were neurological impediment, short stature, disturbed motor skills, pacemaker therapy, and failing Fontan. Accordingly, 4 patients were not accepted to the study. Baseline measurements were made in spring 2018 and final measurements were held in end of year 2018. Prior to training, the patient’s baseline status was measured by body composition, cardiopulmonary exercise test, and muscle strength tests. After the 6-month intervention period, the same measurements were repeated (Fig. 1). All data collection and measurements took place at the Helsinki University Hospital.

### Patient and Public Involvement

The study protocol (Fig. 1) was advocated by the local Ethics committee and accepted by the Pediatric Research Center. Clinical data of 25 patients from cities in Finland were reviewed before contacting the families by phone to outline the study design. Prior to the first visit, the patients and the caregivers were contacted by phone, the willingness to participate was inquired, and if in favor, a signed informed consent form was obtained before enrolling the family to the study. At enrollment, the study protocol was brushed up with the patient and caregiver, and the patient’s disposition and opinion were factored in when self-exercises were tailored. After baseline measurements, two patients failed to follow the study protocol and withdrew from the study. The final study population consisted of 16 children or adolescents of 8–18 years of age (Table 1).

**Table 1:** Patient characteristics

	(*n* = 16)
Age (years)	14.5 ± 2.6
Height (cm)	160.4 ± 11.3
Weight (kg)	51.4 ± 14.4
Gender (f/m)	6/10
Body mass index kg/m^2^	21 ± 3
Medications	
None, ASA, Enalapril	2/12/2
Dominant ventricle of RV-morphology	11
HLHS	11
Dominant ventricle of LV-morphology	5
DILV, RV hypoplasia, TA + TGA, PAIVS	2/1/1/1
Age at Fontan (years)	2.9 ± 0.5
Patient fenestration at admission to study	1

Data are presented as mean ± 1SD

*ASA* Acetylsalicylic acid, *HLHS* hypoplastic heart syndrome, *DILV* double inlet left ventricle, *RV* right ventricle, *TA* tricuspid atresia, *TGA* transposition of the great arteries, *PAIVS* pulmonary atresia with intact ventricular septum

### EUROFIT Tests

The patients performed a EUROFIT test [10] for balance, coordination, and muscle strength with European reference values for children and adolescents aged 9–17 years. The strength of the legs, abdominal muscles, hip flexors, and upper body was measured with squats, sit-ups, and push-ups. The number of repetitions in 30 s was recorded. Standing long jump was used to measure the explosive power of the legs and time spent for a 10 times 5-m shuttle run test evaluated running speed and coordination compared to patients’ own baseline tests.

### Cardiopulmonary Exercise Testing (CPX)

Cardiopulmonary exercise testing was performed using a bicycle spiroergometer (Ergoselect 200P, Ergoline GmbH, Bitz, Germany) with pediatric pedals. The ramp of resistance was determined and increased at 1 min steps 15 W/1 min or 20 W/1 min according to the height of the individual. The exercise was continued until the respiratory quotient (RQ) was at least 1.0 and the subjective level 17–19/20 on the Borg scale for perceived exertion. Work rate was expressed as the peak work rate (Watt) as well as % of the predicted value according to Harkel et al. [11] Arterial O_2_ saturation was assessed non-invasively with two pulse oxymeters (MysignS oximeter, Envitec GmbH, Wismar, Germany).

Breath-by-breath gas analysis was performed using Vyntus CPX (Carefusion 234 GmbH, Hoechberg, Germany). For measurement of respiratory gases, a tightly attached face mask (Rudolph series 7910, Hans Rudolph, Kansas City, MI, USA) was used; the dead spaces of the used mask were added to the program. The ventilatory anaerobic compensation threshold was assessed at the point of slope change of *V*′CO_2_ exceeding *V*′O_2_, increase of *V*′*E*/*V*′O_2_ compared to *V*′*E*/*V*′CO_2_, and increase of PetO_2_ versus PetCO_2_ (partial pressures of O_2_ and CO_2_ in expiratory air) [12].

### Body Composition

Bioelectrical impedance analyses (BIA) were used to estimate patients’ fat percentage and fat-free muscle mass [13]. The body BIA measurement was made in a fasted state after a visit to the toilet with a Biacorpus rx4000 meter suitable for pediatric patients (measurement data based on established data exploited by BodyComposition Version 9.0 Professional software, MediCal HealthCare GmbH, Germany [14]). For calculations, the combined muscle mass of the legs was indexed against the patient's weight, both expressed as kilograms.

### Exercise Prescription

At admission, patients received a questionnaire previously used in children when investigating habitual physical activity and the risk factors of coronary artery disease (the Finnish LASERI study) [15]. To further evaluate the patients’ subjective exercise tolerance, we added the following questions in addition to the standard ones: (1) Can you keep the same running pace as your classmates or teammates? (2) Do you get breathless more easily during sport than your classmates or teammates?

The initial interview and measurements were used as guide for planning the individualized exercise program. The 6-month exercise prescription included a home-based workout program and a daily step goal. The latter was set according to the patient’s maximal oxygen uptake (VO_2max_) value in the baseline tests, which was compared to the mean of the steps in the healthy age group [16].

For the home-based workouts, 80 instructional videos with varying intensity level and focusing on the lower body were filmed and uploaded to a cloud server. Each patient received a personal file with an exercise program consisting of 6–8 exercises to be performed 1–2 times a week. For progressivity, the workout program was upgraded every 2 months. Each patient received a wrist-held ZeFit4activity tracker (MyKronoz, Switzerland) and filled in a diary to record the number of daily steps, workouts, and other physical activity during the day. The weekly summaries from the activity tracker were extracted and the diaries were reviewed at the end of the study.

### Statistical Analysis

The statistical analyses between the baseline and after the 6-month intervention period were calculated using nonparametric *t*-tests. The Mann–Whitney test was used to compare continuous variables between left and right systemic ventricle. Correlations were assessed with Pearson’s correlation coefficient between VO_2_ max and daily amount of activity, lower limb muscle mass, 10 × 5 m shuttle run, standing broad jump, and squats. All data were presented as mean ± standard deviation, and statistical significance was set at *p* < 0.05. The statistical analyses were performed using GraphPad Prism (Prism 8, version 8.4.2).

## Results

### Physical Activity Before Exercise Intervention

According to the LASERI questionnaire, 75% of subjects exercised at least 30 min once a week. Almost all subjects (80%) felt able to participate in all the sport activities they wanted and one third of subjects (31%) regularly engaged in guided exercise, such as a sports club, at least once a week. Compared to their peers, 68% of the subjects found running more difficult and slower. The most important factor-limiting performance was shortness of breath, reported by 31% of subjects. Overall, most subjects (93%) felt themselves healthy and energetic or mostly healthy and energetic. At entrance to the study, patients on average reported weekly physical activity of 3 ± 1.5 h.

**Fig. 2:**
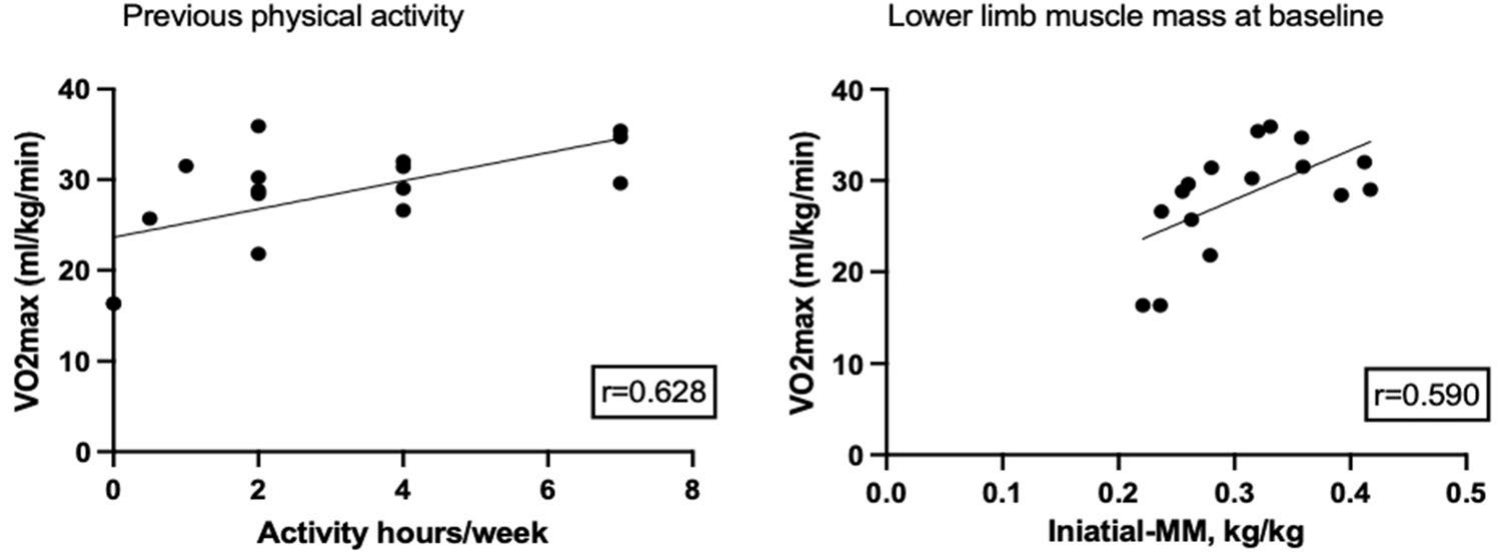
Correlation between VO_2max_ and weekly physical activity (*r* = 0.628, *p* = 0.009)**,** and correlation between VO_2max_ and weight indexed muscle mass of the legs (*r* = 0.590, *p* = 0.016) at baseline in 16 pediatric patients

The baseline investigations demonstrated a statistically significant correlation between weekly physical activity and VO_2max_ (*p* < 0.0091, Pearson *r* = 0.628) (Fig. 2A). In addition, the muscle mass of the legs also demonstrated statistically significant correlation with the results of VO_2max_ (*p* < 0.0161, Pearson *r* = 0.590) (Fig. 2B).

In the cardiopulmonary exercise test, all patients exceeded a respiratory quotient value of 1, which was interpreted as the maximum test. Maximal oxygen consumption (VO_2max_) was 28.3 ml/kg/min ± which is 61 ± 11% of the height- and gender-adjusted reference value [11]. The maximum heart rate (86% of age maximum, 205–0.5 × age) and load endurance (77% of expected reference) were also lower than normal (Table 2) [11].

**Table 2:** Impact of 6-month exercise program on cardiopulmonary exercise capacity and muscle power in juvenile patients with Fontan circulation

	Baseline (*n* = 16)	After 6 months (*n* = 16)	*p*
Body composition			
Lower limb muscle mass (kg/kg)	0.3 ± 0.06	0.3 ± 0.06	0.867
Cardiopulmonary exercise testing			
RQ	1 ± 0.08	1 ± 0.08	0.878
Heart rate max (bpm)	167 ± 16	169 ± 15	0.394
Blood pressure max (mmHg)	164 ± 28	175 ± 26	0.055
VO_2max_ (l/min)	1.4 ± 0.4	1.5 ± 0.4	0.159
VO_2max_ (ml/kg/min)	28 ± 5.9	29 ± 7.2	0.268
AT (ml/kg/min)	18 ± 3.5	20 ± 4.8	0.007
Peripheral O_2_ saturation at peak exercise (%)	92 ± 3.6	93 ± 3.3	0.349
Maximum workload (W)	119 ± 39	132 ± 44	< 0.0012
VE/VCO_2_ slope	32.8 ± 7.6	30.0 ± 5.0	0.049
EUROFIT			
Standing broad jumps (cm)	154 ± 33	160 ± 35	0.073
Squats (reps in 30 s.)	20 ± 4.2	23.7 ± 3.8	< 0.001
10 × 5 m shuttle run (s)	20.3 ± 3.2	20.4 ± 3.9	0.668

*AT* anaerobic threshold; *BP* systolic blood pressure; *HR* heart rate; VO_2max_ maximal oxygen uptake

### Impact of 6-Month Exercise Prescription

**Fig. 3:**
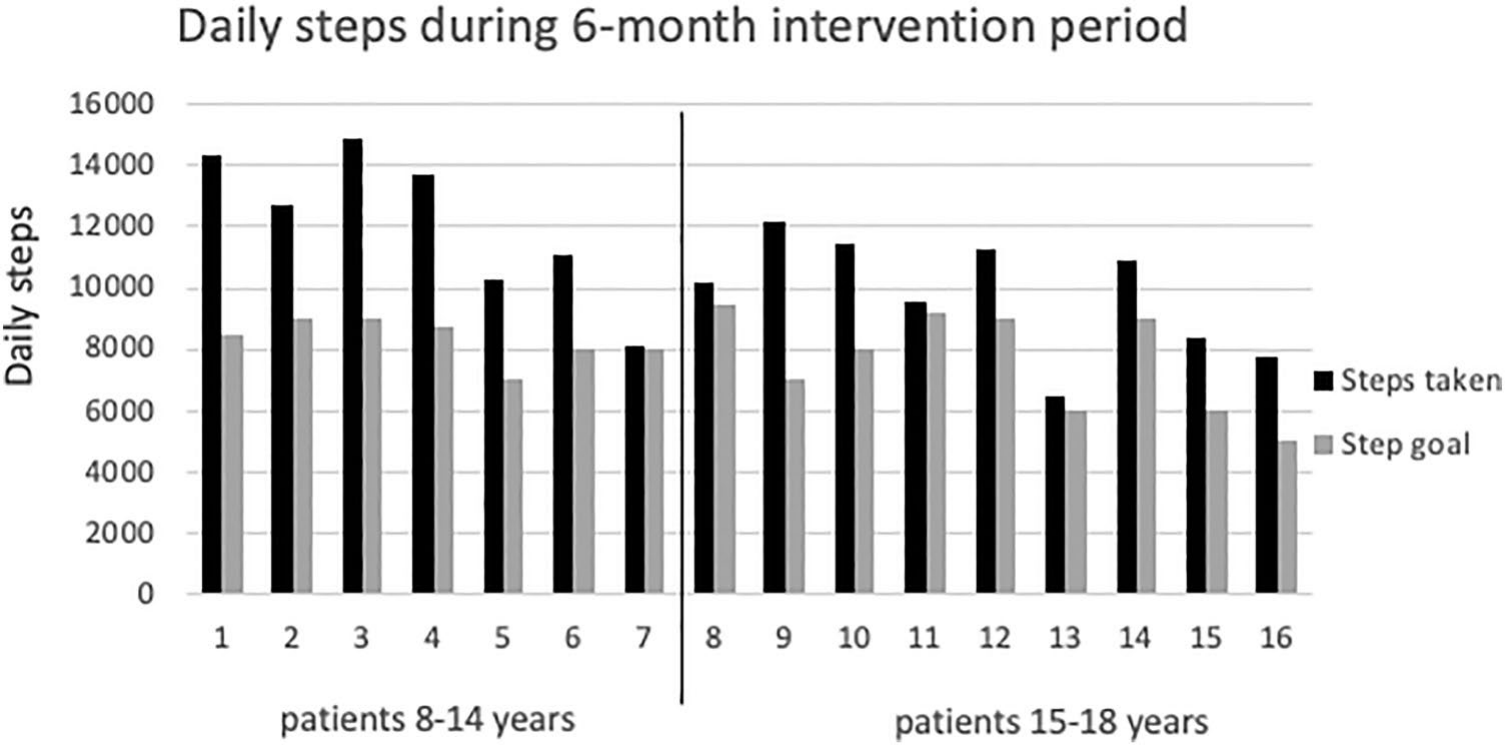
Daily step goal and averaged amount of actual daily steps in 16 Fontan patients who completed the 6-month training period. The vertical line divides patients into two groups by age (patients 1–7 aged 8–14 years; patients 8–16 aged 15–18 years)

Sixteen patients successfully completed the 6-month training period. There were no major adverse events during the exercise intervention, and all measurements were completed without complications. However, 30% of patients reported fatigue that occasionally affected their ability to concentrate at school. During the 6-month study period, all patients exceeded their daily step goal *p* < 0.001 (Fig. 3). There was no statistically significant difference in daily steps between the 8–14 and 15–18 age groups (12,197 ± 2458 vs 9793 ± 1910 steps/day, *p* = 0.071) or for the frequency of domestic strength and coordination training (0.9 ± 0.5 vs 1.5 ± 0.7 workouts/week, *p* = 0.192). In addition to their scheduled program, patients exercised 4 ± 2.6 additional hours per week.

After the 6-month intervention period, VO_2max_ and peripheral O_2_ saturation had remained unchanged at 29.4 ± 7.2 ml/kg/min and 93 ± 3 %, respectively (*N* = 16). However, the anaerobic threshold increased to 20 ± 4.8 ml/kg/min (*p* < 0.0079) and maximum workload to 132.4 ± 44.4 W (*p* < 0.0012, Table 2). In addition, the VE/VCO2 slope also decreased from the baseline (32.8 ± 7.6 vs 30.0 ± 5.0, *p* = 0.049) (Table 2).

At initial measurements, the results from standing broad jump and 10 × 5 m shuttle run were 41% and 62% of reference values, respectively [10]. After 6 months of training, only squats demonstrated significant improvement (*p* < 0.001) (Table 2). At baseline testing, standing broad jump and 10 × 5 shuttle run test correlated with VO2max (*r* = 0.613 and *r* = 0.630, respectively) and following the 6-month intervention period, all three leg muscle tests correlated with VO_2max_ significantly. The correlation between lower limb muscle mass and VO_2max_ was sustained after the intervention period.

## Discussion

Our study demonstrated that the 6-month exercise intervention in pediatric Fontan-operated patients increased weekly physical activity and improved submaximal cardiopulmonary performance, workload tolerance and lower limb strength.

Fontan surgery aims at producing hemodynamic conditions that provide the child with competence for natural daily life activities at the expense of limited reserves of cardiac output. However, when the Fontan circulation works well, it allows in active young subjects a better maximal oxygen uptake [17], and in some offers almost equal performance compared to their healthy peers [18]. Consistently, our data on exercise intervention are encouraging and suggest that the patients were able to gain an improved capacity for the daily grind.

Recent exercise studies to improve the performance of Fontan patients have included endurance and resistance training programs and combination of these two. The duration of interventions has varied from 6 weeks to 6 months, and the intensity of training at 60–90% of the VO2max. Some workout programs have been carried out under a controlled environment and some have been done at home and depended on the patient’s own motivation [19–21]. Despite these efforts, the results have been modest in terms of improving the peak oxygen consumption or lung function [10] Corroborating with these previous observations, we found that in our study patients, having fat and lean muscle mass in the normal range, the 6-month exercise program had an insignificant effect on VO_2max_, which remained at 61–63% of age-matched reference value [22].

Previous observations in young adult patients have demonstrated that peripheral muscles promote Fontan circulation and that indexed systemic flow correlates with lower leg muscle mass [23]. Accordingly, our patients received an individualized exercise prescription at an intensity level of 40–60% of their VO_2max_ including exercises for endurance, lower limb muscle strength, and resilience of the musculoskeletal system. We tested the impact of the multifaceted exercise intervention by cardiopulmonary exercise testing [24], bioimpedance measurement to estimate lower limb muscle mass [25], and EUROFIT test to measure dynamic and explosive muscle strength, speed, and coordination [10]. At initial presentation, previous physical activity, lower limb muscle mass, standing broad jumps, and 10 × 5 m shuttle run showed statistically significant correlations with VO_2max_. Following the 6-month exercise prescription, all these tests and squat repeats per 30 s correlated with VO_2max_.

Maximal oxygen consumption, exercise duration, and maximum load attained during cardiopulmonary exercise test predict prognosis of heart failure patients in biventricular circulation [12, 26]. In the present exercise study, the VO_2max_ remained unchanged, but the workload attained by the test objects significantly increased, and the VE/VCO_2_ slope decreased. Meanwhile oxygen consumption is an important surrogate of cardiac output the VE/VCO_2_ slope is a surrogate for cardiopulmonary capacity since it is a measure of ineffective ventilation [27]. The causes of a high VE/VCO_2_ slope are poor fitness, increased dead space ventilation, ventilation-perfusion mismatching, and an enhanced chemosensitivity-associated ventilation change [28]. Accordingly, reduction of the VE/VCO_2_ slope during the study may indeed indicate physical fitness and improvement in ventilation control and peripheral circulation.

Increased cardiopulmonary performance during exercise may play a role in pulmonary vascular remodeling by facilitating transpulmonary flow, thereby impacting on Fontan hemodynamics. Accordingly, it should be investigated whether the child’s natural physically active lifestyle correlates with a well-functioning Fontan circulation, or whether hemodynamic factors after Fontan completion are more important in designating gratification gained from exercise. In addition to a cardiopulmonary or 6-min walk test [24, 29], we believe that lower limb muscle fitness tests and bioimpedance measurements are useful and cost-effective ways to assess the physical condition of Fontan patients. More studies are needed to investigate the potential benefits of a physically active lifestyle on the development of long-term Fontan outcome.

## Limitations

Based on previously published data on pediatric patients we anticipated that to detect any response to a regular exercise protocol would call for an extended time span during which confirming adherence may be difficult. We appreciated that in the pediatric age group the parents’ contribution in fulfilling the exercise targets is expected to be significant. To improve quality of the data gathered we gave every patient a diary with spaces for categorized entries, a wrist-held activity tracker with a feature of tabulating daily data and asked the families for permission for motivation contacts by telephone. Our method also allowed evaluation of the hours on the go of the test subjects which demonstrated delightful activation in comparison to the habits before the test period.

As commonly observed, our patients showed chronotropic incompetence [30], which persisted, and an only minor improvement in peak blood pressure despite exercise intervention. With the improved submaximal cardiopulmonary performance and reduced VE/VCO_2_ slope, we speculate that our intervention positively influenced ventricular filling, which may have derived from better transpulmonary flow. During the 6 months, the pediatric patients showed natural growth. However, we saw insignificant changes in proportional body fat content and lower extremity muscle mass, or standing broad jump and shuttle run as functional variables. Despite some patients becoming exhausted from the exercises, all individuals achieved the daily step goal set for them. Accordingly, we expect that any demotivation during intervention should have attenuated our findings of improvement. Finally, some of our patients may have ventured technically better after performing the test once already and being self-confident after the training period.

## Conclusions

We have demonstrated that in pediatric patients with Fontan circulation, an individually planned and multifaceted exercise prescription increases endurance and performance without adverse effects. Our study demonstrated that physical activity and lower limb strength correlate with maximum VO_2_ and that regular physical activity steps up submaximal exercise performance. Our findings suggest that in single ventricle patients, appropriate peripheral muscle mass boosts cardiopulmonary performance, which may be of great importance for maintaining function and postponing chronic complications. We believe that Fontan patients should receive exercise counseling from medical professionals to support well-being and commonplace performance, since everyday activities are often in the aerobic range.
