# Correction to: Exercise Prescription Enhances Maximal Oxygen Uptake and Anaerobic Threshold in Young Single Ventricle Patients with Fontan Circulation

**DOI:** 10.1007/s00246-022-02871-7

**Published:** 2022-03-16

**Authors:** Henri Pyykkönen, Otto Rahkonen, Nadja Ratia, Sini Lähteenmäki, Heikki Tikkanen, Päivi Piirilä, Olli Pitkänen-Argillander

**Affiliations:** 1 Faculty of Health Sciences School of Medicine, University of Eastern Finland, Kuopio, Finland; 2 Department of Pediatrics, Helsinki University Central Hospital and University of Helsinki, Helsinki, Finland; 3 Department of Internal Medicine, Helsinki University Central Hospital and University of Helsinki, Helsinki, Finland; 4 Unit of Clinical Physiology of the HUS Medical Diagnostic Center, Helsinki University Central Hospital and University of Helsinki, Helsinki, Finland

## Correction to: Pediatric Cardiology 10.1007/s00246-021-02806-8

In the original article the authors last and given names have been mixed.

This has been corrected in this erratum.

The correct the author names are given below:

- Nadja Ratia
- Sini Lähteenmäki
- Heikki Tikkanen
- Päivi Piirilä
- Olli Pitkänen-Argillander

The original article has been corrected.

